# Outcomes of the rotating hinge knee in revision total knee arthroplasty with a median follow-up of 6.2 years

**DOI:** 10.1186/s12891-021-04205-9

**Published:** 2021-04-07

**Authors:** Jake von Hintze, Mika Niemeläinen, Harri Sintonen, Jyrki Nieminen, Antti Eskelinen

**Affiliations:** 1 Coxa Hospital for Joint Replacement, and Faculty of Medicine and Health Technologies, Tampere University, Niveltie 4, 33520 Tampere, Finland; 2 Department of Public Health, University of Helsinki, Helsinki, Finland

**Keywords:** Revision total knee arthroplasty, Knee replacement, Rotating hinged knee, Hinged knee, Patient-reported outcome measures, Health-related quality of life

## Abstract

**Background:**

The purpose of this study was to determine the mid-term clinical, radiographic and health-related quality of life (HRQoL) outcomes and define the survival rate in patients who had undergone revision total knee arthroplasty (TKA) using the single rotating hinged knee (RHK) design.

**Methods:**

Between January 2004 and December 2013, 125 revision TKAs were performed at our institution using the single RHK implant. We conducted both a retrospective analysis of prospectively collected outcome data of these patients and a prospective follow-up study of all 39 living patients (41 knees). The follow-up phase included an optional extra follow-up visit, PROM questionnaires, and plain radiographs.

**Results:**

The ten-year Kaplan-Meier survival rate of the revision RHK knees was 81.7% (95% CI 71.9–91.6%) with re-revision for any reason as the endpoint. Overall, 15 knees (12% of the total) underwent re-revision surgery during the follow-up. The median follow-up was 6.2 years (range, 0–12.7 years) post-operatively for the baseline group. One mechanical hinge mechanism-related failure occurred without any history of trauma or infection. At the time of the final follow-up, the majority of patients evinced a fairly good clinical outcome measured with patient-reported outcome measures and none of the components were radiographically loose.

**Conclusion:**

We found that in patients undergoing complex revision TKA, fairly good functional outcome and quality of life can be achieved using an RHK implant. Further, it seems that in this type of patient cohort, revision TKA using an RHK implant relieves pain more than it improves ability to function. The NexGen® RHK design can be regarded as a suitable option in complex revision TKA.

**Supplementary Information:**

The online version contains supplementary material available at 10.1186/s12891-021-04205-9.

## Background

In recent years, the incidence of total knee arthroplasty (TKA) has increased worldwide [1–4]. Some studies have predicted that the number of TKA procedures and the subsequent revision burden may increase further in future, which emphasizes the importance of the successful outcome of revision TKA [5–7].

Instability is one of the most frequent causes of knee revision and re-revision along with aseptic loosening and infections [1, 2, 4, 8, 9]. Revision of TKA implants with varying levels of constraint are available to secure knee stability after revision. In the revision or re-revision of TKA patients, the surgeon may confront severe perioperative deformities as well as bony and/or ligament deficiencies. To achieve adequate stability and ideal final outcome, these complex clinical situations may require hinged knee implants. Hinged TKA designs are also used in knees with impaired extensor mechanism, ligamentous laxity producing painful hyperextension of the knee or in patients undergoing oncologic surgery [10–12].

Historically, aseptic loosening occurred more often with hinged knee implants that prohibited rotational motion. These models caused much unwanted stress at the prosthesis-bone interface or on the implant itself, leading to implant failure [10, 11]. To prevent such failures, contemporary hinged knee implant designs allow rotational motion. The range of rotation and how weight is transmitted through the knee depends on the type of hinged knee implant [10].

In the literature, recently published rotating hinged knee implant studies are difficult to compare because of their heterogeneity [11, 13]. Indeed, rotating hinged knee studies are often differentiated by indications (aseptic/septic cases), type of implant, or cohort patients (primary/revision). More clinical evidence is therefore needed on the safety and durability of these commonly used knee replacements in revision knee arthroplasties.

The purpose of this study was to determine the mid–term clinical, radiographic, and health-related quality of life (HRQoL) outcomes in patients who underwent revision TKA using the single rotating hinged knee (RHK) design at our institution between January 2004 and December 2013.

### Study design and methods

From January 2004 to December 2013, 125 revision TKAs were performed at our institution using the NexGen® RHK implant. The study site is an academic high-volume tertiary referral center with an annual volume of approximately 150 revision TKAs.

This study comprised three phases: first, a prospective follow-up study of all the surviving patients of this cohort was conducted. Second, a retrospective analysis of the prospectively collected outcome data recorded into the electronical joint replacement database at our institution was carried out. Third, information on possible revision surgeries that might have been performed elsewhere, and thus not recorded into our own database, was cross-checked from the Finnish Arthroplasty Register.

All RHK revision knee arthroplasties were performed using the medial parapatellar approach and a tourniquet was also routinely used. TKAs were carried out under spinal anesthesia in combination with intravenous sedation. General anesthesia was only used if there was a contraindication to spinal anesthesia. Immediate, full weight-bearing was allowed, and all patients were mobilized on the first postoperative day. An antithrombotic prophylaxis with low-molecular-weight heparin, enoxaparin, was administered for 4 weeks postoperatively. All details of perioperative care and possible complications were recorded in the hospital’s electronical database in a routine manner. In 48 knees (38%), the patella was resurfaced in the revision TKA. In 25 knees (20%), the patella had already been resurfaced in the primary TKA operation. Cemented femoral and tibial components were used in all operations, and the choice between cemented and uncemented stems was based on surgeon preference. The trabecular metal cones were used in 13 cases (10%). At the baseline of the study, 119 patients (125 knees) were included in the retrospective analysis, where all the demographics, surgery reports, first post-operative visits (at 2–3 months), possible post-operative complications, and adverse events as well as reasons for revisions were obtained from the medical records and the hospital’s electronical clinical database. Information on possible revision surgeries performed on patients elsewhere who were lost to follow-up (18 patients, 18 knees) was cross-checked from the Finnish Arthroplasty Register [4].

**Fig. 1:**
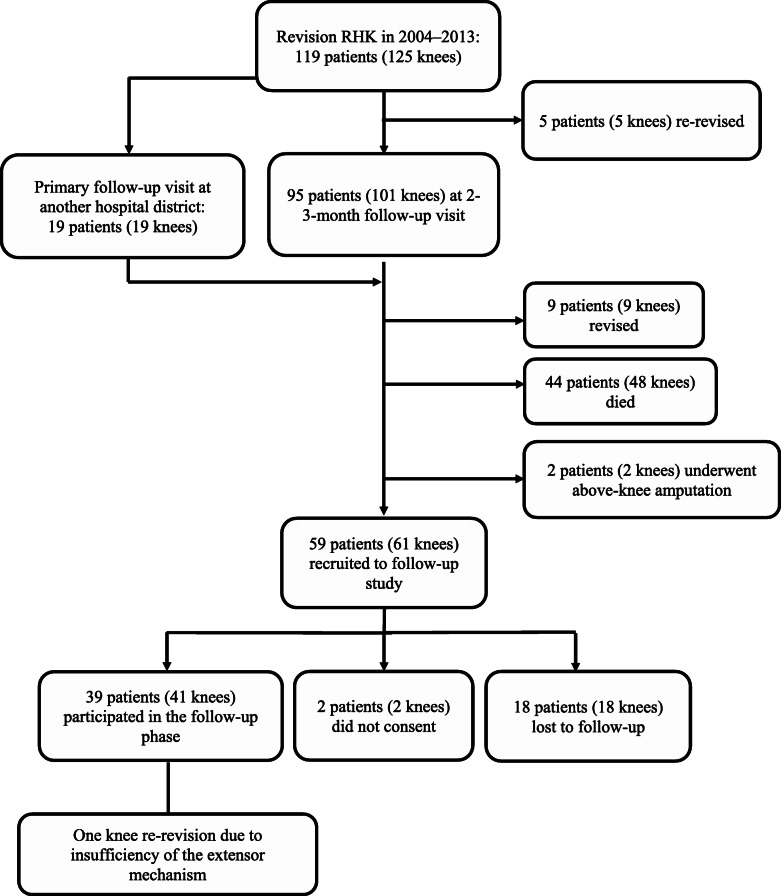
Flowchart describing the selection and loss to follow-up of the study participants

In the prospective study phase, an extra follow-up visit was scheduled between 4 and 14 years post-operatively, depending on the year of the index operation. All of the 59 living and unrevised patients (61 knees) were recruited by telephone for an extra follow-up visit at our outpatient clinic (Fig. 1). In total, 39 patients (41 knees) agreed to participate in the follow-up phase of this study. Those patients who were unable to attend the extra follow-up visit received a set of PROM questionnaires by surface mail and were asked to visit their nearest health care provider for plain radiographs to be taken. The extra follow-up visit included plain radiographs of the operated joint, clinical assessment by a physiotherapist, and the use of PROMs, i.e., the Oxford Knee Score (OKS), the Knee Injury and Osteoarthritis Outcome Score (KOOS), the 15D (generic measure of health-related quality of life), and the Forgotten Joint Score (FJS).

### Radiographic evaluation

#### Preoperative

Radiographic findings, such as osteolytic defects and stability of the patella, were evaluated from the pre-operative radiographs. Pre-revision bone defects were classified according to Anderson Orthopaedic Research Institute (AORI) classification [14] and these classifications were compared to peroperative findings for final assessment. The stability of the patella was examined from the skyline patellar radiographs. All patient records and pre-operative radiographs were examined by the first author who had not been involved in the revision surgeries and had not met the patients during the follow-up visits. To estimate the reliability of the measurements, a sample (*n* = 20) of the pre-operative radiographs was later reviewed by an experienced orthopaedic surgeon who was blinded to the original measurements.

#### Postoperative

All postoperative plain radiographs from the extra follow-up were evaluated by two senior orthopaedic surgeons (co-authors JN and AE). Radiographic evaluation was performed from standardized weight-bearing antero-posterior (AP), lateral, and skyline patellar views. Radiographs were assessed for the presence of radiolucent lines or osteolytic defects.

### Patient-reported outcome measures

Health-related quality of life (HRQoL) was measured using the comprehensive generic 15D instrument. The instrument combines the advantages of a profile and a preference-based single index measure. The 15D instrument includes the following 15 dimensions: mobility, vision, hearing, breathing, sleeping, eating, speech, excretion, usual activities, mental function, discomfort and symptoms, depression, distress, vitality, and sexual activity. For each dimension, the respondent chooses one of the five ordinal levels that best describe their state of health at the time (best =1; worst =5). The single index score (15D score) represents the overall HRQoL on a 0–1 scale (1 = full health, 0 = being dead). The dimension level values reflect the goodness of the levels relative to no problems on the dimension (=1) and to being dead (=0). These values are then calculated from the health state descriptive system (questionnaire) by using a set of population-based preference or utility weights. Mean dimension level values are used to draw 15D profiles for groups [15]. The minimum clinically important change or difference in the 15D score has been estimated to be ±0.015 on the basis that people can, on average, feel such a difference [16].

We compared our study population’s 15D results to those of an age- and gender-standardized sample of the general Finnish population (*n* = 4052) taken from the Health 2011 Survey carried out by the National Institute for Health and Welfare of Finland [17].

The OKS and the KOOS have been widely used to assess the outcomes of knee replacements [18, 19]. In this study, the OKS was categorized into four different grades: poor (0–26), fair (27–33), good (34–41), and excellent (42–48) [18]. The KOOS is an extension to the WOMAC Osteoarthritis index and includes five separately scored subscales. The subscales are Pain, other Symptoms, Function in daily living (ADL), Function in sport and recreation, and knee-related quality of life [20]. Forgotten Joint Score (FJS) assesses the patient’s ability to forget the replaced joint while performing recreational activities and in daily life. A higher degree of ¨forgetting¨ the joint indicates a better outcome of surgery [20].

The Kaplan-Meier (K-M) analysis was performed to assess the survival rate of the RHK implant. Both survival rates and 95% confidence intervals (CI) were derived from K-M models. The independent samples t-test was used to test the statistical significance of the differences in the mean 15D results between the groups. Statistical significance was set at *p* < 0.05. The statistical analysis was performed with SPSS Statistics for Mac (version 25.0). Competing risk analysis was performed with R (version 4.0.2). The study was funded by an institutional grant from Zimmer Biomet Inc. (Warsaw, IN, USA). The study was approved by the local ethical committee (R17010).

## Results

The baseline study group consisted of 86 women (91 knees) and 33 men (34 knees) with a median age of 71.7 years (range, 31–95 years) at the time of revision TKA. Five females and one male patient underwent bilateral revision TKA (RHK on both sides). In the final follow-up group, most of the patients were women (72%, *n* = 28/39). The median follow-up was 7.3 years (range, 4.0–12.7 years) at the time of the final follow-up. Demographics, revision indications, and pre-revision bone defects are summarized in Table 1. The most important indication for revision was instability (*n* = 54, 43.2%). Pre-revision bone defects were mostly grade 1 both on the tibial (*n* = 87, 69.6%) as well as on the femoral side (*n* = 90, 72.0%) according to the AORI classification.

**Table 1:** Demographics of study population

	The baseline		Final follow-up	
N of knees (patients)	125	119	41	39
Age (median, range)	71.7	31.2–95.3	67.1	42.0–85.1
Body mass index (median, range)	29.3	18.8–52.0	30.6	23.1–43.0
Follow-up, years (median, range)	6.2	0.0–12.7	7.3	4.0–12.7
Females (knees, %)	91	73%	30	73%
Number of previous knee surgeries (median, range)^a^	2	1–6	2	1–5
Indications for revision RHK (knees), N (%)				
Instability	54	43.2%	22	53.7%
Prosthetic joint infection	26	20.8%	6	14.6%
Loosening, wear and osteolysis	20	16.0%	3	7.3%
Periprosthetic fracture	11	8.8%	3	7.3%
Complication of the extensor mechanism^b^	7	5.6%	2	4.9%
Malalignment	6	4.8%	4	9.8%
Arthrofibrosis	1	0.8%	1	2.4%
Stability of patella preoperatively, N (%)				
Stable	66	52.8%	21	51.2%
Subluxation	11	8.8%	3	7.3%
Chronic dislocation	11	8.8%	5	12.2%
Fragmentation/Demineralization	4	3.2%	2	4.8%
Girdlestone/Spacer/Flap	4	3.2%	1	2.4%
Fracture	3	2.4%	1	2.4%
Instability of knee, N (%)				
Antero-Posterior	47	37.6%	15	36.6%
Medio-Lateral	82	65.6%	28	68.3%
Pre-revision bone defects (AORI, N (%))^c^				
Tibia				
grade 1	87	69.6%	30	73.2
grade 2a	12	9.6%	5	12.2%
grade 2b	11	8.8%	3	7.3%
grade 3	15	12.0%	3	7.3%
Femur				
grade 1	90	72.0%	34	82.9%
grade 2a	10	8.0%	2	4.9%
grade 2b	9	7.2%	1	2.4%
grade 3	16	12.8%	4	9.8%

^a^including the index primary operation and possibly the spacer procedure

^b^including patellar ligament ruptures and impaired extensor mechanism

^c^According to Anderson Orthopaedic Research Institute (AORI) classification.

### Clinical outcome and PROMs in the final follow-up group

OKS was good or excellent in a slight majority of patients (51%, 21 knees), moderate in 6 knees, and poor in 14 knees at the time of the extra follow-up visit. The median OKS was 29 (*n* = 33, range, 8–48). The median KOOS for Pain was 75 (*n* = 34, range, 19–100), Symptoms 73 (*n* = 33, range, 18–100), ADL 69 (*n* = 34, range, 7–100), Sport/Rec 18 (*n* = 33, range, 0–100), and QOL 44 (*n* = 33, range, 6–100) at the time of final follow-up. The median FJS was 33 (*n* = 34, range, 0–100).

**Fig. 2:**
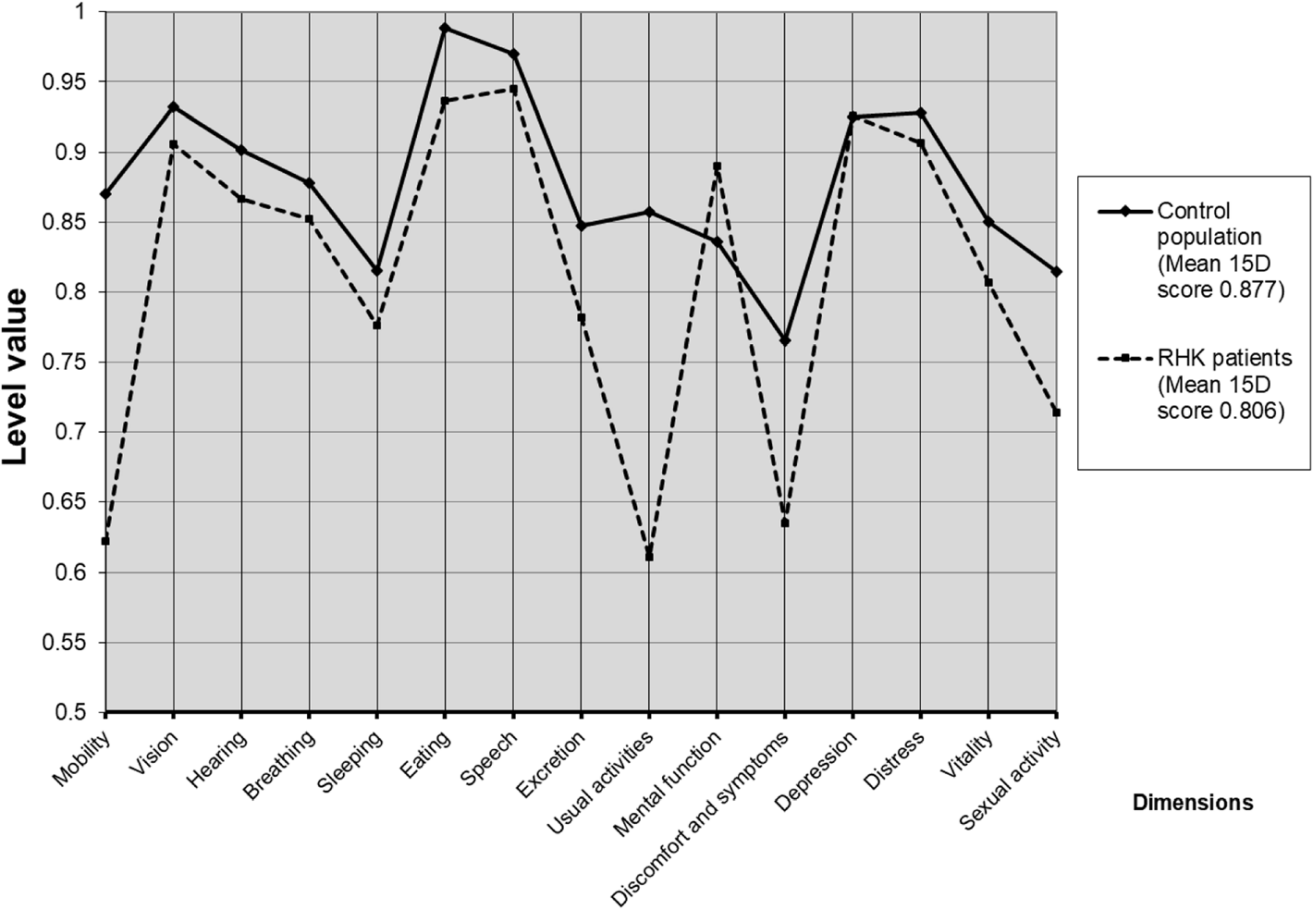
Comparison of mean 15D profiles between control population and RHK group

The mean 15D score of the patients was 0.806 (range, 0.523–1.000) at the time of the final follow-up. The age- and gender-standardized control population average was 0.877 (range, 0.778–0.943). This difference is both clinically important and statistically significant (*p* = 0.002). The mean level values of the dimensions of the patients compared to those of the age- and the gender-standardized general population are shown in Fig. 2.

On average, there was a trend for RHK patients to be better off than the control population on the dimension of mental function, but the difference was not statistically significant (*p* = 0.098). RHK patients were, however, statistically significantly worse off than the control population on the dimensions of mobility (*p* < 0.001), usual activities (*p* < 0.001), and discomfort and symptoms (*p* = 0.008).

At the time of the final follow-up, plain radiographs were available for 37 patients (39 knees). One patient (1 knee) had a radiolucent line in the cement-bone interface next to the medial condyle of the tibia; components were stable and no osteolysis was seen. Two patients (2 knees) had mild radiolucencies around the proximal part of the femoral stem, and one of them also had distal pedestal bone formation, which is typical for uncemented stems. None of the components were radiographically loose.

### Revisions, survival rate and complications in the baseline group

In total, 15 knees (12% of the total) underwent re-revision surgery during the follow-up (Table 2). In eight cases, the reason for re-revision surgery was prosthetic joint infection (PJI). One mechanical hinge mechanism-related complication led to re-revision surgery: both the tibial cone and also the anterior part of the femoral hinge-post had fractured in that knee (See details in Table 2).

**Table:** Table 2

Revisions	Failure	Time to failure	Treatment
1	Periprosthetic femoral fracture	6.2 years	Osteosynthesis combined with exchange of the femoral component and the polyethylene insert
2	Aseptic loosening (femur)	7.2 years	Exchange of the femoral component and the tibial insert
3	Prosthetic joint infection	0.17 years	DAIR
4	Arthrofibrosis	5.8 years	Open lysis of adhesions through medial parapatellar arthrotomy
5	Mechanical hinge-related complication (without trauma); fracture of the tibial cone and the anterior part of the femoral hinge-post	3.0 years	Exchange of the tibial insert and the hinge mechanism
6	Patellar dislocation (traumatic)	0.2 years	Secondary patellar resurfacing
7	Rupture of the quadriceps tendon and the MPFL^a^	5.1 years	Knee arthrodesis
8–15	Prosthetic joint infection	9 days–8.7 years	2-stage revision arthroplasty

^a^ Patient had multiple complications, see Additional file 1

*MPFL* The medial patellofemoral ligament

*DAIR* Debridement and implant retention

**Fig. 3:**
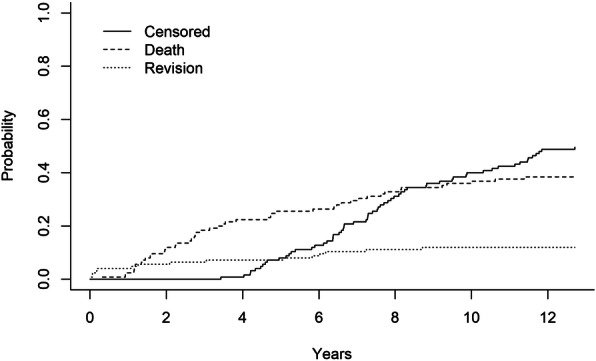
Cause-specific probability and overall survival probability of arthroplasty revision and death as competing events

The ten-year K-M survival rate for the revision RHK implant was 81.7% (95% CI 71.9–91.6%) with revision for any reason as the endpoint. The ten-year K-M survival rate was 82.4% (95% CI 72.6–92.2%) when those revisions, in which only patellar resurfacing was performed, were excluded. Competing risk analysis for overall survival probability of arthroplasty (40.0% at 10 years) and probability of re-revision (12.0% at 10 years) and death (36.8% at 10 years) as competing events is shown in Fig. 3.

During the follow-up, 31 postoperative complications were recorded (25%; Additional file 1). Later on, one of these patients also underwent knee arthrodesis due to severe extensor mechanism insufficiency 5.1 years after the index operation (marked with A in Table 2 & Additional file 1). Two other patients also had multiple complications during the follow-up (marked with B and C in Additional file 1). Two above knee amputations had to be performed: one patient underwent amputation because of critical limb ischemia (ASO) and the other one because of chronic PJI that was difficult to keep in control. These have been considered as the endpoint of survival (Additional file 1).

## Discussion

In our study, we demonstrated a fair mid-term clinical outcome and also acceptable implant survivorship in patients undergoing complex revision arthroplasty using the NexGen® RHK implant. A slight majority of the patients reported good or excellent OKS at the time of the final follow-up. Moreover, 39% (*n* = 13/33) of the patients described their knee pain as either non-existent or very mild. However, HRQoL was worse in these patients than in the control population as measured with the 15D instrument. The ten-year K-M survival rate was 81.7% with revision for any reason as the endpoint. Overall, 15 knees (12% of the total) underwent re-revision surgery during the follow-up, and the most common reason for re-revision was prosthetic joint infection (PJI; 8/15 of the cases). One mechanical failure of the hinge mechanism led to re-revision. None of the RHK implants were radiographically loose at the time of the final follow-up.

We acknowledge a few limitations in our study. First, the number of participants was low in the final clinical follow-up visit, which reduces the generalizability of our results. Medical comorbidities and overall fragility preventing patients from participating in this kind of clinical follow-up study were the main reasons given for the low participation rate. However, this is an obvious universal problem when conducting research on frail, elderly patients. Moreover, previous follow-up studies have also reported difficulties in achieving a complete follow-up for this challenging patient group [21–24]. By cross-checking the patient’s re-revisions from the Finnish Arthroplasty Register, however, we were able to make sure that we had captured all the re-revisions performed on these patients and also those performed outside our hospital district on patients who were lost to our clinical follow-up. Second, the indications for knee revision surgery were variable, as both aseptic and septic revisions were included (21%) and comorbidities were partly unknown and therefore not considered in this study. Third, the preoperative PROMS were unavailable, which made it difficult to evaluate the influence of RHK arthroplasty on symptoms, ability to act, or quality of life. Moreover, there was lack for information of stem lengths and the use of augments.

We consider that our study also has a few strengths. As far as we know, this current study is one of the largest series evaluating single rotating hinge knee implant model outcomes only in revision TKA. Moreover, the median 6.2 years follow-up can be considered satisfactory. To our knowledge, this study, which evaluated the efficacy of a single implant on revision TKA, has the largest cohort and longest follow-up for the NexGen® RHK implant in the published literature [11, 21–33].

The final follow-up group reported variable OKS results. It must be noted, however, that a slight majority reported good or excellent OKS, but the total median score was 29, which can be categorized as fair [18]. Our OKS results are in accordance with previous studies [26, 33]. Böhler et al. reported an OKS of 29 (mean, *n* = 26) for revision arthroplasty in five-year mid-term follow-up for different single rotating hinge knee implants [33]. Furthermore, Giurea et al. reported an OKS of 30 (mean, *n* = 62) after two-year follow-up [26].

Helito et al. investigated single rotating hinge knee implants prospectively and reported the KOOS after one-year follow-up. The study included only nine patients, however, and six of them were primary rotating hinge knee implants, and therefore it is difficult to compare them with KOOS [34]. We are unaware of any previous study that has reported the KOOS after revision RHK arthroplasty with mid-term follow-up. Our cohort’s KOOS subscales reflect a good outcome for treated pain and symptoms during the follow-up.

There is a paucity of reported FJS in rotating hinge knee patients. Röhner et al. studied single hinge knee implants in primary arthroplasty and reported an FJS of 39 in the mean 20-months follow-up [35]. This is slightly less than our reported FJS. However, our study cohort comprised only revision knees, and thus a meaningful direct comparison between the results was not possible.

The mean 15D score of our RHK cohort was statistically significantly and clinically importantly worse than in the control population. The patients were worse off, especially on the dimensions of mobility, usual activities, and discomfort and symptoms. It is reasonable therefore to assume that these kinds of complex revision arthroplasty patients have difficulties when compared with the age- and gender-standardized general population. Further, RHK patients were better only on the dimension of mental function. However, the difference was not statistically significant and this might be attributable to selection bias. Patients with severe neurodegenerative diseases were lost to follow-up and this could have biased our HRQoL results. Miettinen et al. reported a retrospective study, in which QoL was assessed the 15D instrument before and after primary TKA, but the type of implants remained unknown. After TKA, mean 15D score was 0.870 (*n* = 731) at the time of 12 month follow-up [36]. This is markedly higher than the mean 15D score (0.806) in our study at the time of final follow-up visit. However, a meaningful direct comparison between these results is not possible for various reasons.

Kouk et al. published a review study of rotating hinge implants for revision TKA. They evaluated studies which included more than 50 rotating hinge implants used in the revision setting. They summarized complication rates to have been between 7 and 63% in the previous studies [13].

In this regard, our postoperative complication rate (25%) bears comparison with the current literature. In the current study, infections were a notable part of all the complications 29% (*n* = 9/31) and of all the revisions 60% (*n* = 9/15). Moreover, 32% (*n* = 10/31) of all complications were related to extensor apparatus complications. It should be noted that only two of these had a preoperative well-functioning extensor mechanism (Additional file 1). These complications can be devastating and have a major influence on the ability to act [37]. Kearns et al. examined the same single RHK design (14 primary and 65 revision patients) and reported postoperative complications in 39% of patients, which included three (13% of all postoperative complications) mechanical hinge failures and five (21%) extensor mechanism ruptures. Two patients had failure of their hinge-post and one had a fracture of the hinge mechanism [21]. This is more than the one mechanical failure reported in our study.

The 10-year implant survival rate of 81.7% is comparable with the rates reported in other rotating hinge knee studies. In their meta-analysis that included 12 different studies, Yoon et al. compared the survivorships and outcomes of RHK and condylar constrained knee prostheses. The rotating hinge knee implant models were variable. The meta-analysis revealed an overall survival rate of 81.3% for all RHK implants at the mid-term (5–10 years) follow-up [38].

## Conclusion

To conclude, we found the NexGen® RHK design to be a suitable option in complex revision TKA. This implant provided fairly good functional outcome and quality of life in our cohort of patients, and the mid-term implant survivorship was acceptable in this challenging patient group. Also mechanical problems in the hinge mechanism were rare. However, Nexgen RHK should only be used in complex cases, as complications are still frequent, and the results of this design are comparable, not superior, to those previously published of other rotating hinge knee designs.

## Supplementary Information

**Additional file 1.** Postoperative revision surgeries.

## Data Availability

The datasets generated and/or analysed during the current study are not publicly available due General Data Protection Regulation at our hospital district but are available from the corresponding author on reasonable request.

## Abbreviations

HRQoL: Health-Related Quality of Life
TKA: Total Knee Arthroplasty
RHK: Rotating Hinged Knee
PROM: Patient reported outcome measures
OKS: Oxford Knee Score
KOOS: Knee Injury and Osteoarthritis Outcome Score
FJS: Forgotten Joint Score
AORI: Anderson Orthopaedic Research Institute
PJI: Prosthetic Joint Infection
DAIR: Debridement, Antibiotics and Implant Retention
MPFL: The medial patellofemoral ligament
